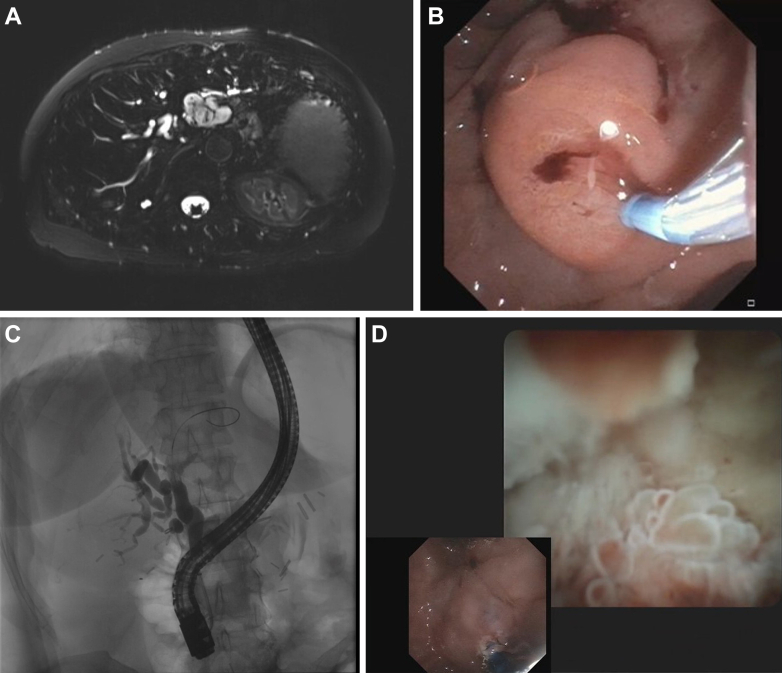# A Compelling Mimic: Fish Mouth Major Papilla and Villous Biliary Mucosa on Cholangioscopy

**DOI:** 10.1016/j.gastha.2026.101005

**Published:** 2026-05-11

**Authors:** Andrew Kelley, Somashekar Krishna, Hamzah Shah

**Affiliations:** 1 Department of Internal Medicine, The Ohio State University, Wexner Medical Center, Columbus, Ohio; 2 Department of Gastroenterology, Hepatology, and Nutrition, The Ohio State University, Wexner Medical Center, Columbus, Ohio

A 62-year-old woman with a history of metastatic low-grade mucinous neoplasm presented with obstructive jaundice. Magnetic resonance imaging of the abdomen with contrast demonstrated mild intrahepatic biliary ductal dilation, a dilated common bile duct, and a lobulated left hepatic lobe lesion (Figure A). An endoscopic retrograde cholangiopancreatography revealed the classic “fish mouth” appearance of the major papilla from which thick, gelatinous fluid was drained (Figure B) and a diffusely dilated bile duct (Figure C). SpyGlass cholangioscopy identified abnormal mucosa with a papillary texture in the common hepatic duct (Figure D). A biliary plastic stent was placed, and biliary patency was achieved. Spybite biopsies of abnormal mucosa in duct bifurcation revealed low-grade appendiceal mucinous adenocarcinoma.

Low-grade mucinous neoplasm is rare with an indolent course. However, when associated with peritoneal involvement, the disease becomes incurable, with a 10-year survival rate of 45%–68%. The “fish mouth” appearance of the major papilla is a characteristic and pathognomonic sign associated with intraductal papillary mucinous neoplasms involving the bile duct. SpyGlass cholangioscopy in biliary intraductal papillary mucinous neoplasms often reveals villous protrusions, intraductal mucin, and targeted biopsies that are frequently diagnostic. We present what may be the most compelling mimic of this condition, with the final diagnosis being metastatic mucinous adenocarcinoma.

**Figure:**